# Cobb Angle Reduction in a Nearly Skeletally Mature Adolescent (Risser 4) After Pattern-Specific Scoliosis Rehabilitation (PSSR)

**DOI:** 10.2174/1874325001711011490

**Published:** 2017-12-29

**Authors:** Marc Moramarco, Kathryn Moramarco, Maja Fadzan

**Affiliations:** Scoliosis 3DC, 3 Baldwin Green Common, Suite 204, Woburn, MA 01801

**Keywords:** Scoliosis-specific exercise, Pattern-specific scoliosis rehabilitation, Adolescent idiopathic scoliosis, Schroth method, Schroth Best Practice

## Abstract

**Introduction:**

It has long been said that exercise-based rehabilitation for scoliosis is ineffective, however, these reports studied general exercises. This case report is a prospective one-year follow-up of a nearly skeletally mature adolescent female (Risser 4) with idiopathic scoliosis treated with Pattern-Specific-Scoliosis Rehabilitation (PSSR).

**Methods:**

The 15-year old patient recommended for surgery (initial Cobb angle of 45°) completed a 16-hour scoliosis-specific back school (according to Schroth Best Practice^®^), over the course of five weeks. She continued with her program at home, and followed up with the lead author after 6 months and 1 year.

**Results:**

The patient achieved a 13° reduction in her primary thoracic Cobb angle. Postural improvement and reduction in trunk rotation (ATR) was also achieved (-4° in the thoracic spine, and -5° in the lumbar spine).

**Conclusion:**

Pattern-specific scoliosis rehabilitation (PSSR) works to reduce the asymmetrical load caused by scoliosis. PSSR is effective in stabilizing Cobb angle, and can, in some cases, reduce Cobb angle in adolescents. Patients recommended for surgery may be candidates for conservative treatment. This case suggests that the practice of discontinuing conservative treatment at Risser stage 4 should be re-evaluated.

## 1 INTRODUCTION

Scoliosis is a three-dimensional deformity of the spine and trunk [1, 2]. Adolescent idiopathic scoliosis (AIS) is the most prevalent form (80–90%). Other forms include congenital, neuromuscular, mesenchymal disorders and syndromic scoliosis [3]. A consensus statement by the American Academy of Orthopedic Surgeons (AAOS), Scoliosis Research Society (SRS), American Academy of Pediatrics (AAP) and Pediatric Orthopedic Society of North America (POSNA) recommends screening at ages 10 and 12 for girls and at age 13 or 14 for boys [4]. Currently, thirty-three U.S. states mandate that adolescents be screened for scoliosis [5]. Diagnosing scoliosis and stopping curve progression during the pre-pubertal growth phase is of primary importance. Current evidence demonstrates that progression can be halted successfully during growth in the majority of cases with bracing [6].

When scoliosis is detected in the mild phase, the standard practice is periodic monitoring to wait to see if progression occurs. Despite this, many patients and their families prefer not to be idle and risk eventual progression. For those with moderate scoliosis (25º-45º), bracing is the traditional course of action when the patient has significant growth potential (Risser 0-2) [7]. At the mid-forty to fifty-degree Cobb angle range and beyond, most surgeons recommend surgery [7].

Scoliosis can be detected at any phase. It is not uncommon for scoliosis to go undetected until it is moderate or even severe. This is for a variety of reasons, for example, double major curves (two curves of relatively equal magnitude) can sometimes go unnoticed since the trunk appears balanced. As a general rule, the greater the curve magnitude at the time of diagnosis, the more likely that progression will occur [8, 9].

Long-term studies have shown that untreated AIS is relatively benign [10], but that is not to imply that scoliosis management should be neglected. Surgical intervention comes with many risks [11], therefore, there is a need for an effective, non-invasive approach. With scoliosis, there is an impairment of spinal and rib cage mobility as a result of asymmetric loading [12]. This impairment, for some, may have a negative influence on respiratory function and vital capacity and may contribute to psychological distress due to torso asymmetry and/or pain [13-16].

The Schroth method, used in Europe for nearly a century, addresses scoliosis according to curve pattern. The goal is to enable patients to work to counteract the asymmetric loading on the spine and trunk [17]. This is accomplished mainly via a proprietary corrective breathing technique, which contributes to mobilizing and stabilizing the spine and rib cage. The patient learns the necessary skills for independent practice to attempt to prevent curve progression or to improve scoliosis and overall health and function [18].

There is growing evidence in support of the method. Its benefits include Cobb angle stabilization – with the potential for reduction in some adolescents, and improvement of angle of trunk rotation (ATR) [18-23]. This can translate to an improvement of postural symmetry. Improvements in muscle strength, pulmonary function, chest expansion, cosmetic appearance, self-esteem and curve reduction have also been documented [18-32].

Schroth treatment offers the potential for curve reduction for some skeletally immature children and adolescents. This is because the immature spine is more amenable to conservative treatment due to remaining growth potential [33]. However, treatment need not be limited to adolescents since there are inherent benefits for patients of all ages. This case report is a prospective short-term follow-up (one year) of a nearly skeletally mature adolescent female treated with pattern-specific-scoliosis rehabilitation (PSSR).

## 2 MATERIALS AND METHODS

### 2.1 Initial Diagnosis

The subject of this report is a healthy, active fifteen-year old female (at time of initial presentation) with AIS. She reported a maternal aunt with a slight scoliosis. The patient first detected a trunk asymmetry in late fall/early winter of 2015. This prompted a visit to the patient’s primary care doctor. That doctor ordered an x-ray, which was taken on February 16th, 2016. As a result of the x-ray, Cobb angles of 41° thoracic (apex at T9) and 27° lumbar scoliosis (apex at L2) were reported (the upper thoracic curve was not recorded). The patient was immediately referred to an orthopedic surgeon at a local hospital for evaluation.

In April 2016, the patient visited the surgeon who subsequently ordered another full spine x-ray to evaluate the patient’s growth plates and any curve progression. The surgeon also ordered an MRI to rule out any spinal abnormalities or potential causalities due to the apparent rapid onset. The x-ray, performed on April 6, 2016, revealed Cobb angles of 28° upper-thoracic (T1-T6), 43° mid-thoracic (T6-T12) and 20° lumbar (T12-L4). At this point, it was determined that the patient was a Risser stage 4, indicating that she was nearly skeletally mature. This patient began menses at twelve years old and at the time of the April 2016 X-ray was three years post-menarcheal. The MRI performed on April 11, 2016, yielded normal results with no vertebral anomalies or paraspinal soft tissue abnormalities.

### 2.2 Recommendation for Surgery

At that point, the surgeon recommended spinal fusion and instructed the patient to stop cheerleading and tumbling. He warned the patient’s mother that her daughter’s scoliosis could worsen. This claim is substantiated by a study which found that untreated skeletally mature patients with thoracic curves of 30° - 50º progressed an average of 10.2º over forty years [34]. The patient and her mother chose not to return to the surgeon but instead sought evaluation from the lead author on April 14, 2016 because, as the mother stated, she “did not want foreign matter in [her daughter’s] body at such a young age.”

### 2.3 Scoliosis-Specific Back School

The patient’s examination included a complete medical history, visual inspection, palpation, spinal range of motion, neurological and orthopedic testing. The examination yielded the following postural asymmetries: shoulder and pelvic unleveling, left ventral prominence, a right dorsal prominence and a left lumbar prominence on the forward bend test. Palpation revealed mild hypertonicity of the left upper thoracic, right mid/lower thoracic and left lumbar paravertebral musculature. All range of motion, neurological, and orthopedic tests yielded normal results.

Clinical parameters measured initially and at follow-up visits included vital capacity, chest expansion, and angle of trunk rotation (ATR). ATR was determined using a Bunnell scoliometer™ in a forward-bending position at the same locations on the spine – upper thoracic, mid-thoracic and lumbar, all performed by the lead author. Three readings of chest expansion, to measure rib mobility, were measured using a cloth tape measure marked in millimeters. Chest expansion was measured at the junction of the xiphoid process and the body of the sternum. Three readings of spirometry to measure forced vital capacity (FVC) and forced expiratory volume at one second (FEV1) were also performed. The type of spirometer used was the hand-held Contec SP10 (has a +/- 3% margin of error). Photos were also taken initially and at follow-up visits to document trunk asymmetries with the purpose of making postural comparisons over time.

The lead author provides Pattern-Specific Scoliosis Rehabilitation (PSSR) according to Schroth method principles he learned at the Asklepios Katharina Schroth Clinic in Germany. The PSSR program used includes protocols referred to as Schroth Best Practice^®^ – an updated version of the Schroth method for use on an outpatient basis.

The patient completed sixteen hours of a multimodal scoliosis-specific back school program divided into 2-hour sessions, over the course of five weeks. Program components include:

• Curve-pattern specific spinal education
• *Spinal Mobilizations: active, passive and active resisted
• Pattern-specific modified activities of daily living (ADLs)
• Physiologic^®^ exercises: corrective sagittal plane exercises
• 3D Made Easy: 3D exercises combining ADLs and Schroth corrective breathing
• Power Schroth: advanced Schroth method exercises (primarily in the upright position for optimal muscle engagement)
* The patient in this study declined the mobilizations component of the program.

The patient received a customized exercise manual and a video recording of her program. This is done to ensure that patients are confident performing each component of the PSSR independently. This patient was instructed to perform her program daily throughout the follow-up period.

According to the patient and her mother, the patient was fully compliant with her program for the first four months after initial instruction (daily exercise). As of August 2016, the patient began performing her exercises 4 days a week (of her own accord). She is a member of her high school’s fall and winter cheerleading teams, for which she has practice a few times per week. In addition to her cheerleading activities, in early winter 2017, the patient participated in a 5-week CrossFit training program. The patient reported non-compliance with her PSSR for all of February 2017 and part of March 2017. According to the patient’s mother, her daughter was simply “burnt out” from being involved in so many activities. As of mid-March 2017, the patient’s mother reported that her daughter is again semi-compliant with her program, exercising an average of four days per week.

## 3 RESULTS

The patient’s April 2016 x-ray was measured by the lead author as 32° upper thoracic, 45° mid-thoracic and 24° lumbar. This x-ray is used as a baseline to gauge results as it was taken just prior to program commencement. The 1-year follow-up x-ray revealed Cobb angle reduction to 33° upper thoracic, 32° mid-thoracic and 18° lumbar, as measured by the lead author. Alternate measurements were obtained by the attending radiologist and an independent chiropractic radiologist (Table **1**).

At initial evaluation angle of trunk rotation measured 4° upper thoracic, 10° mid-thoracic and 9° lumbar. These measurements reduced to 1º upper thoracic, 6º mid-thoracic, and 4° lumbar at follow-up 1-year later (Table **2**).

## 4 DISCUSSION

The patient in this case report participated in a scoliosis-specific back school, or Pattern-Specific Scoliosis Rehabilitation (PSSR). The program is modeled after the German Scoliosis in-Patient Rehabilitation (SIR) at the Asklepios Katharina Schroth Clinic with recent updates that enable ease of learning and outpatient instruction [18].

In the United States, there has been a long-standing opinion among practitioners that exercise for scoliosis does not help prevent progression or improve a curve. The SRS website states, “there is little evidence to show that physical therapy is more effective than doing nothing in stopping the curve from getting worse during growth” [35]. Historically, very few studies on the topic of exercise and scoliosis have been produced to substantiate this claim, and none in the U.S. have ever studied pattern-specific scoliosis rehabilitation [36].

That said, the patient in this study demonstrated curve reduction of her primary curve and spinal stabilization as a result of PSSR. This is demonstrated in the x-rays b, c and d, Fig. (**1**). Not only did the patient achieve Cobb angle reduction (from 45° to 32°, lead author measurements) but she improved her spinal balance overall. This is demonstrated on the x-ray, which shows that the apex of her curve has moved closer to midline. This occurred despite the patient discontinuing her exercise routine for about six weeks just prior to the final x-ray.

Had the patient done nothing, she may have progressed beyond the 45º level shown in her April 2016 x-ray. This cannot be stated with certainty, but it has been established that curves diagnosed at a greater magnitude are more likely to progress [8]. It may also be extrapolated that because her spine changed for the better, it may have also deteriorated. While there are reported cases of spontaneous reduction in mild scoliosis in immature patients [37], this has not been reported in larger curves.

Notably, at the time of treatment the patient was a nearly skeletally mature fifteen-year-old girl (Risser 4) and three years post-menarcheal. While, in practice, Risser 4 correlates with the cessation of spinal growth in females [38], it has been previously reported that vertebral growth can still occur at Risser stage 4 [39]. This is in contrast to industry practices indicating that patients at Risser 4 are considered skeletally mature and are weaned from brace treatment [6]. In female patients, clinicians often use the marker of two-years post-menarche, but this has also been challenged [40]. Certainly, determining the end-point of growth is a complex undertaking. However, this case demonstrates the potential for scoliosis improvement at Risser 4 to Risser 5 and calls into question whether there are truly “no options” other than surgery for patients close to skeletal maturity.

## 5 PATTERN-SPECIFIC SCOLIOSIS REHABILITATION (PSSR)

Each patient program begins with educational instruction for their unique asymmetric spinal configuration. This enables the patient to better comprehend the concepts taught during the course of instruction. Patients then learn activities of daily living (ADLs) according to curve pattern. Modified static and dynamic postures are taught so patients learn to self-correct and reduce asymmetric loading on the trunk, particularly in the frontal plane [31, 41-43]. Once patients learn how how to modify their postures when sitting, standing, etc. and have internalized this information, with practice, their corrective postures become their new habitual postures. It is important for patients to fully understand the rationale behind their spinal configuration and ADLs so that when a new activity presents itself the patient knows what to do and why. Active, passive, and hands-on active/resisted spinal mobilizations are also taught. Although the patient in this case declined learning mobilizations, the lead author recommends them to re-establish joint mobility, which facilitates postural correction [24]. She did, however, receive spinal manipulations from the lead author throughout the duration of her 5-week program.

Daily exercise components of the program include physiologic^®^, 3-D Made Easy and Power Schroth exercises. The physiologic exercises^®^, introduced in 2006, focus on the sagittal plane [30]. This is because many patients with AIS present with a thoracic hypokyphosis and lumbar hypolordosis, particularly at the apical area [44, 45]. This has been found to be a contributing factor of curve instability and progression [17, 46]. Physiologic exercises^®^ are used to help re-establish normal physiologic curves and improve spinal mobility [30]. Other daily exercises include 3D Made Easy, simple Schroth exercises that combine the scoliosis-specific ADLs and corrective breathing [30]. 3D Made Easy exercises are meant to be used throughout the course of the day. Incorporating Schroth corrective breathing at intervals during the day have a greater impact than a single session of Schroth daily and may be less intrusive in a patient’s life. Lastly, Power Schroth exercises are introduced. These are intensive Schroth exercises, performed primarily in an upright position for optimal muscle engagement [23].

**Fig. (2):**
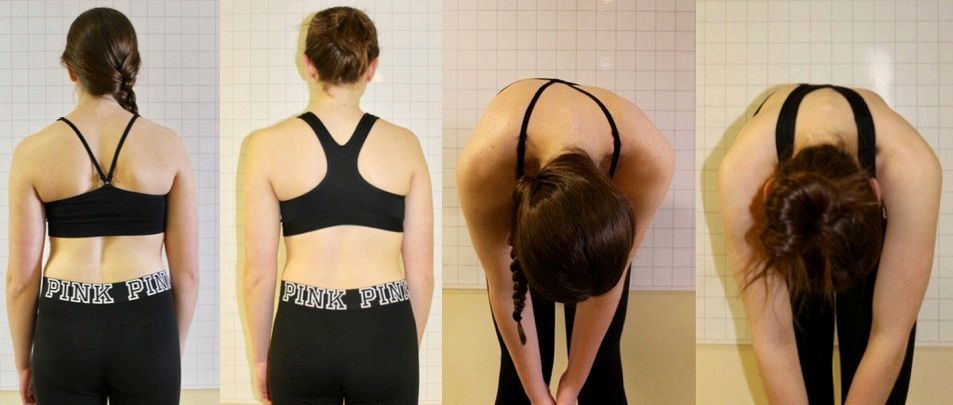
Comparison photos taken at initial examination and at one-year follow-up. Follow-up photos (2nd and 4th images from left) show improved body symmetry and reduced rotation.

The PSSR program focuses on alleviating the asymmetric loading of the spine that occurs with scoliosis [47]. Trunk derotation is a key aspect of Schroth exercises with the goal of improving trunk symmetry and postural appearance [24] (Fig. **2**). At the outset of the program, ATR was measured at three different levels along the spine; the locations were based on the patient’s Schroth curve pattern. Clinically, she demonstrated a substantial reduction in trunk rotation at each level (ATR) and maintained them through her last follow-up (Table **2**).

While improving ATR helps to provide a more symmetric postural appearance, Cobb angle is the marker that clinicians and parents look to most often to monitor scoliosis and for decision making. In this case, the patient’s Cobb angle reduced, but a discussion of Cobb angle would not be complete without pointing out its limitations as well.

Firstly, Cobb angle is a two-dimensional measurement of a three-dimensional condition [48]. A measurement alone does not tell the entire story of a scoliotic spine. Furthermore, there is the issue of intra-observer and inter-observer error, with the latter typically exhibiting greater disparity [49-51]. Cobb angle is formed by the intersection of two lines, one parallel to the end plate of the superior vertebra and the other parallel to the end plate of the inferior end vertebra, above and below the apex, respectively [52]. Disparities may occur because the measuring practitioner chooses the lines of delineation–those of the most tilted vertebrae–which is subjective [53]. Sometimes there can be difficulty in determining these end plates due to poor quality x-ray [54].

When monitoring scoliosis, disagreement may also arise as to whether a ≥5° or ≥10° difference in Cobb angle is considered the marker of true change [51]. That said, a ≥5º change is a fairly accepted standard by most physicians [55]. This patient achieved a >10º Cobb angle reduction of her primary curve, according to the measurements of the lead author, the independent chiropractic radiologist, and the attending radiologist(s) (Table **1**). Since the attending radiologist(s) who measured the four x-rays were all different individuals, these are considered inter-observer measurements and subject to greater discrepancy [56].

Cobb angle measurements are subject to other limitations as well. Varying Cobb angles can result due to different measurement methods (i.e. manual vs. digital) [53, 57]. Other factors may be patient position and diurnal variation [58]. In order to have a perfect comparison, patients should ideally assume identical positioning for successive x-rays and have images taken at a similar time of day [58].

Cobb angle measurements have implications on treatment plans and resulting actions. Recommendations for treatment may vary by practitioner according to curve classification (mild, moderate or severe) and remaining growth potential. Inaccuracies can lead to unnecessary testing, MRI, and anxiety-inducing circumstances for patient and parents. Conflicting opinions from physicians and radiologists regarding measurement can cast doubts on how to proceed with treatment.

According to Weinstein et al. for patients >30º, progression in adulthood is 0.73º annually [16]. Scoliosis curves measuring below 30° at skeletal maturity are less likely to progress [16]. This patient, at 33º, is close to the 30º marker. If she remains committed to her program, the lead author is of the opinion that she can thwart progression risk in adulthood. Considering that the patient is at full fusion of the iliac crest apophysis (Risser 5), she may elect to reduce the exercise component of her program to 3 to 4 times per week.

## CONCLUSION

This case shows an instance where pattern-specific scoliosis rehabilitation resulted in Cobb angle reduction and curve stabilization in an adolescent scoliosis patient. It stands to reason that for some patients with AIS, other treatment options should be explored before considering surgery. Also, the practice of discontinuing conservative treatment at Risser 4 should be re-evaluated.

## Figures and Tables

**Fig. (1):**
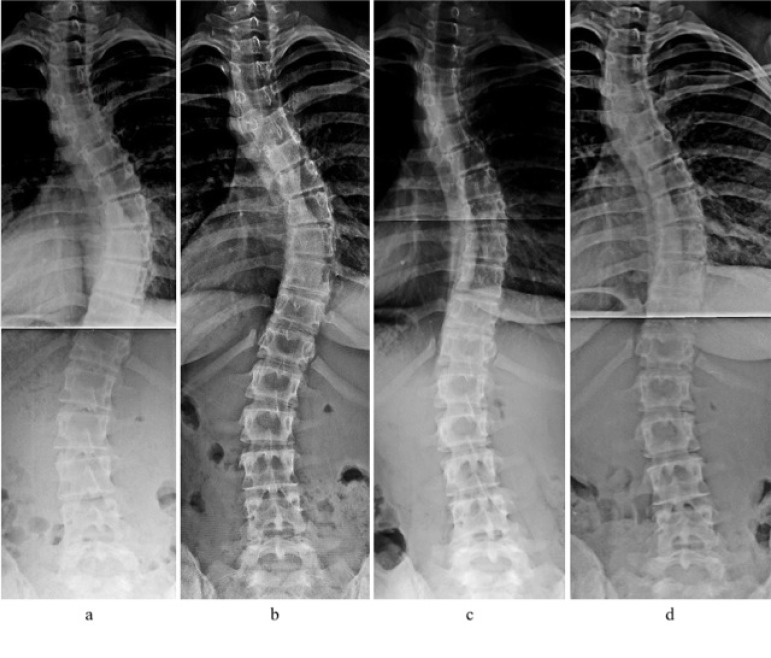
a) February 2016, b) April 2016, c) October 2016, d) April 2017. X-ray b is the patient’s initial x-ray prior to beginning her PSSR program. X-ray d was taken one year later. The patient’s x-rays were performed at one of two local hospitals in the metropolitan Boston area.

**Table 1:** The patient in this case report was x-rayed just before the start of her PSSR program (4-6-2016) and at 6-month and 1-year follow-up. Measurements done by the lead author indicate a 13° reduction in primary Cobb angle, while those done by the attending radiologist(s) indicate a 20° reduction and those done by an independent chiropractic radiologist indicate a 14° reduction.

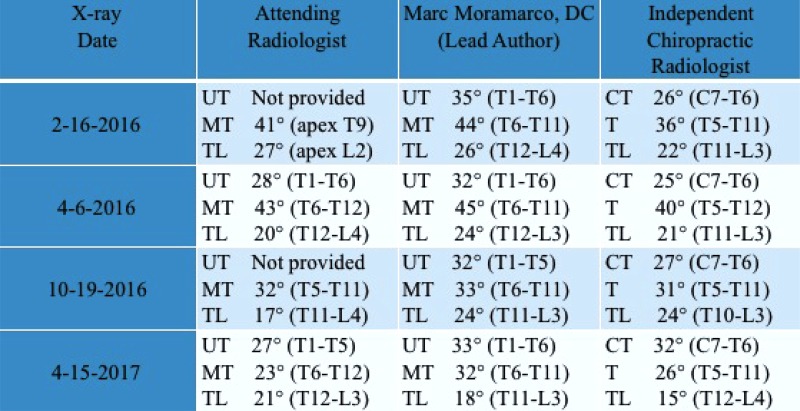

**Table 2:** ATR measurements, done by the lead author, at initial examination, end of program, 6-month follow-up, and 1 year follow-up visits.

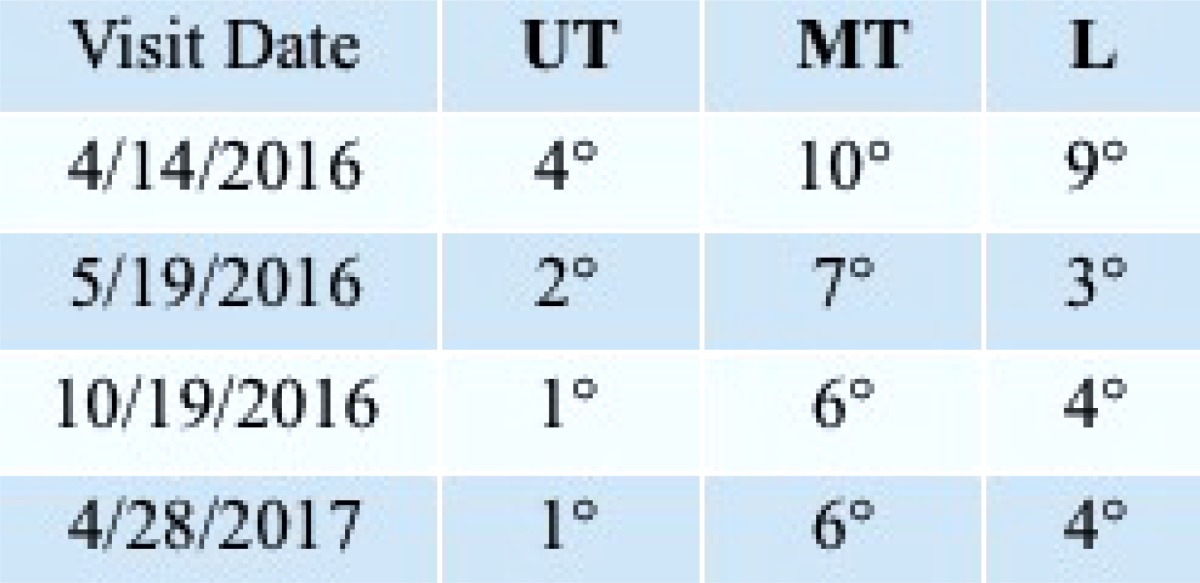

## LIST OF ABBREVIATIONS

AAOS: = American Academy of Orthopedic Surgeons
AAP: = American Academy of Pediatrics
ADL: = activity of daily living
AIS: = adolescent idiopathic scoliosis
ATR: = angle of trunk rotation
POSNA: = Pediatric Orthopedic Society of North America
PSSR: = pattern-specific scoliosis rehabilitation
SRS: = Scoliosis Research Society
